# A patient-specific treatment model for Graves’ hyperthyroidism

**DOI:** 10.1186/s12976-017-0073-6

**Published:** 2018-01-09

**Authors:** Balamurugan Pandiyan, Stephen J. Merrill, Flavia Di Bari, Alessandro Antonelli, Salvatore Benvenga

**Affiliations:** 1 Department of Mathematics, University of Wisconsin - Whitewater, 800 W. Main Street, Whitewater, 53190 WI USA; 2 Department of MSCS, Marquette University, Milwaukee, 53201-1881 WI USA; 3 Divisione di Endocrinologia, Policlinico Universitario di Messina, Messina, 98125 Italy; 4 Department of Clinical and Experimental Medicine, University of Pisa, Pisa, Italy; 5 Divisione di Endocrinologia, Policlinico Universitario di Messina, Messina, 98125 Italy

**Keywords:** Hyperthyroidism, Methimazole, Graves’ disease, Thyroid receptor antibodies

## Abstract

**Background:**

Graves’ is disease an autoimmune disorder of the thyroid gland caused

by circulating anti-thyroid receptor antibodies (TRAb) in the serum. TRAb mimics the action of thyroid stimulating hormone (TSH) and stimulates the thyroid hormone receptor (TSHR), which results in hyperthyroidism (overactive thyroid gland) and goiter. Methimazole (MMI) is used for hyperthyroidism treatment for patients with Graves’ disease.

**Methods:**

We have developed a model using a system of ordinary differential equations for hyperthyroidism treatment with MMI. The model has four state variables, namely concentration of MMI (in mg/L), concentration of free thyroxine - FT4 (in pg/mL), and concentration of TRAb (in U/mL) and the functional size of the thyroid gland (in mL) with thirteen parameters. With a treatment parameter, we simulate the time-course of patients’ progression from hyperthyroidism to euthyroidism (normal condition). We validated the model predictions with data from four patients.

**Results:**

When there is no MMI treatment, there is a unique asymptotically stable hyperthyroid state. After the initiation of MMI treatment, the hyperthyroid state moves towards subclinical hyperthyroidism and then euthyroidism.

**Conclusion:**

We can use the model to describe or test and predict patient treatment schedules. More specifically, we can fit the model to individual patients’ data including loading and maintenance doses and describe the mechanism, *hyperthyroidism*→*euthyroidism*. The model can be used to predict when to discontinue the treatment based on FT4 levels within the physiological range, which in turn help maintain the remittance of euthyroidism and avoid relapses of hyperthyroidism. Basically, the model can guide with decision-making on oral intake of MMI based on FT4 levels.

**Electronic supplementary material:**

The online version of this article (doi:10.1186/s12976-017-0073-6) contains supplementary material, which is available to authorized users.

## Background

**Fig. 1:**
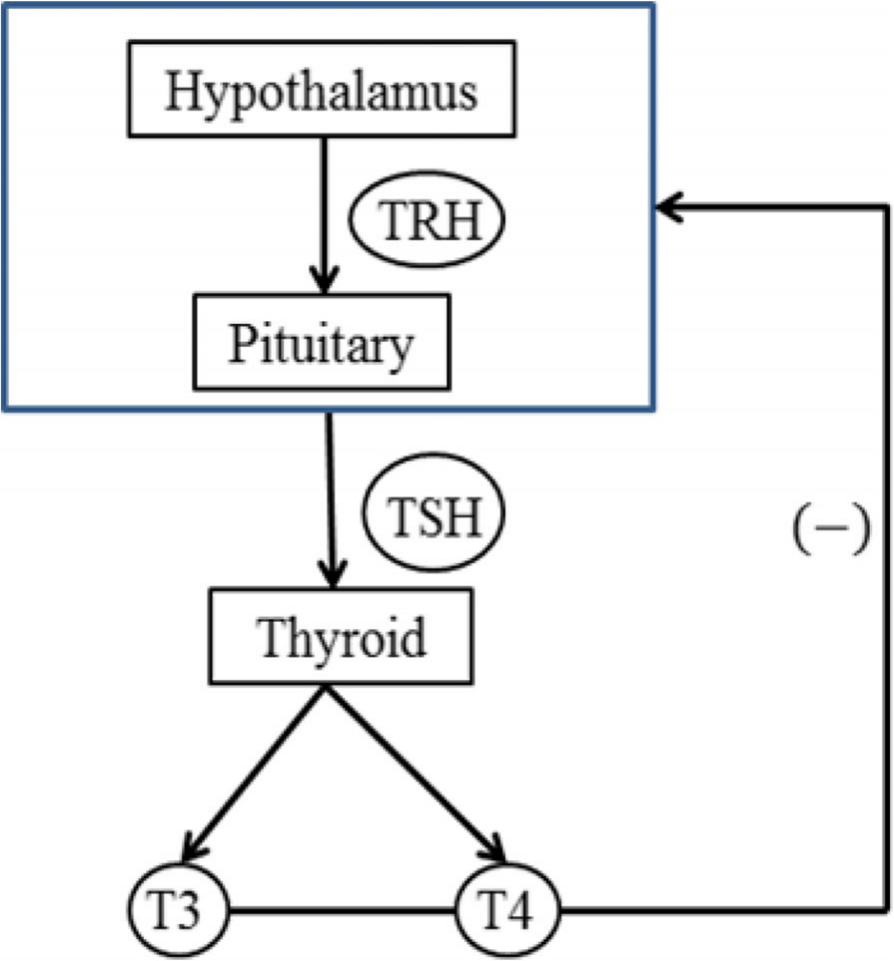
The HPT axis. A negative feedback loop of the hypothalamus-pituitary-thyroid (HPT) axis is shown in this picture. The thyroid stimulating hormone (TSH) stimulates the thyroid gland to produce and secrete the hormones, triiodothyrnoine (T3) and thyroxine (T4), which in turn inhibits the production of TSH. A minus sign indicates the negative feedback

Thyroid stimulating hormone (TSH) stimulates the thyroid follicular cells by binding to the TSH receptors (TSHR) which activates the production and secretion of thyroid hormones, thyroxine (T4) and triiodothyronine (T3) into the serum. Too much T4 and T3 inhibits the production of TSH, which forms a negative feedback loop (Fig. 1). Anti-thyroid stimulating receptor antibodies (TRAb) sometimes produced by the immune system that circulates in the serum and mimics the action of TSH [1, 2]. As a result, TSH goes below lower reference range and almost undetectable in the serum but TRAb continuously stimulates the gland to produce and secrete hormones, which induces the overactive thyroid gland (hyperthyroidism) in normal individuals [3]. This autoimmune problem is called Graves’ disease. Graves’ disease is characterized by the loss of immunotolerance to thyroid antigens and the development of TRAb due to a complex interaction between genetic and environmental factors [4, 5]. It is classified as an organ-specific autoimmune disease; however other organs such as the eye, the pituitary, the skin and the joints involved in the disease process because of the presence of TSH receptors within these organs might be targeted by TRAb [6].

The incidence rate of Graves’ disease was reported to be 20−30 cases per 100,000 individuals each year [7, 8]. Sex difference plays an important role in autoimmunity especially in Graves’ disease [9] and there is high risk on women aged between 40−50 years [1, 2]. The clinical presentation of Graves’ disease includes symptoms, physical findings and hyperthyroidism. The symptoms may include anxiety, difficulty in sleeping, fatigue, weight loss, palpitations and eye swelling. The physical findings may include diffuse goiter, increased pulse pressure, tremor, warm moist palms and tachycardia. Graves’ hyperthyroidism can be categorized as overt or subclinical. Overt hyperthyroidism is defined as extremely low serum concentrations of TSH and high serum concentrations of thyroid hormones, T4 and T3 [10]. Subclinical hyperthyroidism is defined as low serum TSH, but normal serum T4 and T3 concentrations. In the United States, the prevalence of hyperthyroidism is approximately 1.2*%* (0.5*%* overt and 0.7*%* subclinical); the most common causes include Graves’ disease, toxic multinodular goiter and toxic adenoma [11].

The laboratory hallmark of Graves’ hyperthyroidism is finding elevated levels of serum free thyroid hormones, free T4 (FT4) and free T3 (FT3), associated with undetectable serum TSH and positivity for serum TRAb at one point in time [12]. Approximately, the normal reference range for FT4 is (7−18) pg/mL, FT3 is (23−50) ng/L and TSH (0.4−4.0) mU/L respectively [13]. Graves’ hyperthyroidism is generally treated with one of the three approaches based on patients’ choice, namely use of antithyroid drugs (thionamides) to restore euthyrodism - normal levels of thyroid hormones, destruction of thyroid via radioactive iodine or removal of thyroid via surgery. Antithyroid drugs can be used as primary treatment and can be used as pretreatment in selected patients prior to radioactive iodine therapy or prior to surgery. So, this article focuses on antithyroid drug treatment method with Methimazole (MMI), which results in the clinical progression of the patients from hyperthyroidism to euthyroidism.

No perfect curative medical treatment exists for Graves’ disease; however one can control the signs and symptoms of hyperthyroidism with the help of Methimazole (MMI). Depending upon the severity of patients’ hyperthyroidism, the starting dose of MMI is normally given between 10 to 60 mg per day [1], which can be continued for about 12−18 months until patients achieve euthyroidism. The amount of MMI can be given as a single daily oral dose or divided doses to achieve normalization of thyroid function. MMI has the benefit of once-a-day administration and a reduced risk of major side effects [11]. After a single oral dose, the rate of absorption of MMI from the gut to serum happens rapidly and differently in hyperthyroid patients, so the peak serum concentration of MMI reaches within 30 to 180 min [14–16]. After the initiation of treatment, the levels of FT4 and FT3 should be monitored periodically [17] and the doses should be lowered or increased in accordance to the measured levels. In this treatment, MMI can act as an inhibitor of thyroid hormone synthesis by interfering with the action of thyroid peroxidase (TPO) enzyme in the gland. Thereby, thyroid hormone secretion gradually decreases and patients become euthyroid over time, which is the goal of treatment (see Fig. 8). The mechanism of this treatment procedure can be simply described as *hyperthyroidism* →*euthyroidism*.

Typically, higher doses of MMI is prescribed when patients are in severe hyperthyroidism; for example serum FT4 levels are greater than 3 times more than the upper reference range of FT4. If higher doses are maintained for a long time period or given when patients do not have a severe hyperthyroidism, there is a possibility for overshooting during treatment. The mechanism of overshooting can be described as *hyperthyroidism* →*hypothyroidism*. Similarly, lower doses of MMI are administered sometimes during treatment because of the variability within individuals, so there is a possibility for undershooting and that mechanism can be described as *hyperthyroidism* →*subclinical hyperthyroidism*. After stopping the MMI treatment, relapse of hyperthyroidism may occur for some patients with Graves’ hyperthyroidism. Relapse is totally independent of the type of drug and dosage administered but linked to restoration of euthyroidism [18]. Relapse mechanism can be described as *euthyroidism* →*hyperthyroidism*. Some patients exhibits exacerbation which means returning to hyperthyroidism while still on MMI treatment, as opposed to relapse (which is return hyperthyroidism after withdrawal of MMI) [19]. In this article, we explain all of the possible mechanisms involved in the Methimazole treatment through a parameter from our model.

Although the initial dose of MMI is based on FT4 levels and decided by the treating physician, the maintenance dosages of MMI are tricky and challenging during the time course of treatment. An appropriate maintenance dosage depends on frequent observation of the patient, which makes the treatment difficult in some sense and dependable on patient follow-up. With the model, we can test or control FT4 levels within the physiological range of (7−18) pg/mL with appropriate maintenance dosages for the entire time period of treatment. We can also predict or describe the relapse of hyperthyroidism after discontinuation of MMI treatment.

## Methods

### Construction of a model

We present a patient-specific treatment model to describe the effect of MMI treatment in patients with Graves’ hyperthyroidism. Basically, the model describes the clinical progression from hyperthyroidism to euthyroidism in accordance to treatment procedure. An earlier model constructed by Langenstein et al. [20] for Graves’ hyperthyroidism did not keep track of MMI treatment over time. However, their article was an inspiration and provided a base for this new work. So, we build our model with the following key assumptions and state variables.

**Fig. 2:**
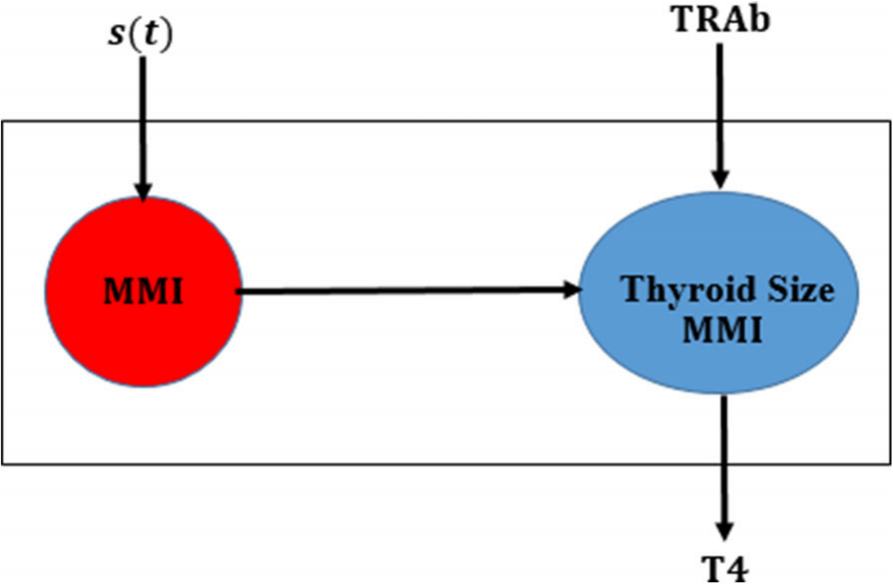
A lumped compartmental model of blood and thyroid gland. We assume Methimazole (MMI) dynamics in the blood serum is similar to MMI dynamics in the functional thyroid gland. The release of thyroxine (T4) depends on the stimulation of TRAb and inhibition of MMI in the functional gland

Assumptions:

1. TRAb mimics TSH and stimulates the thyroid follicular cells to grow, produce and secrete thyroid hormones [1].
2. After oral administration, Methimazole (MMI) absorption occurs rapidly and almost completely from gut to blood serum and its bioavailabity is estimated to be approximately 93% [21, 22].
3. Thyroid intakes Methimazole (MMI) from the blood serum, which in turn inactivates the functional growth of the gland [23].
4. A portion of Methimazole (MMI) drains out from the blood serum after intake [21, 24].
5. The functional size of thyroid gland is a hidden compartment in the model [20, 25, 26].
6. After oral administration, the blood serum concentration of Methimazole (MMI) exhibits a similar dynamical pattern as intrathyroidal concentration of MMI [22, 27] (see the support for this assumption on page 449−450 in the article [22] and Fig. 2).

State Variables:

- *x*(*t*)= the amount of MMI (mg) per liter of blood serum at time *t*.
- *y*(*t*)= the amount of FT4 (pg) per milliliter of blood serum at time *t*.
- *z*(*t*)= the functional size of thyroid gland (mL) or the volume of proportion of active cells at time *t*.
- *w*(*t*)= the amount of TRAb (U) per milliliter of blood serum at time *t*.
- *s*(*t*)= the amount of MMI orally taken per day per liter of body volume (*mg*/*L*/*day*).

The model is given below with initial conditions.

1 $$\begin{array}{*{20}l}\frac{dx}{dt}&=s(t)-\frac{(k_{1}z)x}{k_{a}+x}-k_{2}x & x(t_{0})=x_{0},  \end{array} $$

2 $$\begin{array}{*{20}l} \frac{dy}{dt}&=\frac{(k_{3}z)w}{k_{d}+w}-k_{4}y & y(t_{0})=y_{0}, \end{array} $$

3 $$\begin{array}{*{20}l} \frac{dz}{dt}&=k_{5}\left(\frac{w}{z}-N\right)-k_{6}zx & z(t_{0})=z_{0}, \end{array} $$

4 $$\begin{array}{*{20}l} \frac{dw}{dt}&=k_{7}-\frac{k_{7}x}{k_{b}+x}-k_{8}w & w(t_{0})=w_{0}  \end{array} $$

where *x*(*t*)≥0,*y*(*t*)≥0,*z*(*t*)>0, *w*(*t*)≥0 and the initial conditions *E*_0_=(*x*_0_,*y*_0_,*z*_0_,*w*_0_).

In Eq. (1), the first term contains the time-dependent function *s*(*t*), which represents the rate of change of MMI dosing at time *t*. The rate of change of MMI dosing is assumed to be zero when no MMI is taken orally. The middle term, $-\frac {(k_{1}z)x}{k_{a}+x}$, represents the uptake rate of MMI by the thyroid gland with maximal saturation rate (*k*_1_*z*). We modeled the uptake rate with Michealis-Menten kinetics as supported by the literature [28]. The last term, −*k*_2_*x*, represents the excretion or elimination rate of the drug through non-specific mechanism.

In Eq. (2), the first term, $\frac {(k_{3}z)w}{k_{d}+w}$ accounts for the secretion rate of free thyroxine, which we modeled through the Michaelis-Menten kinetics with the maximum secretion rate as (*k*_3_*z*). The second term, −*k*_4_*y*, represents the elimination rate of free thyroxine from the blood.

In Eq. (3), the first term, $\left (\frac {w}{z}-N\right)$, represents the growth rate of the functional thyroid gland in the presence of TRAb in the blood wherein *N* indicates the maximal growth ratio. The second term, −*k*_6_*zx*, represents the inactivation rate of thyroid functional size by the MMI treatment.

In Eq. (4), the first term, *k*_7_, represents the maximum production rate of TRAb due to autoimmune response. The middle term, $-\frac {k_{7}x}{k_{b}+x}$, represents the inhibition rate of TRAb due to MMI dosing. We further assume the the maximum inhibition rate is the maximum production rate of TRAb. The maximum inhibition rate will be reached asymptotically as the concentration of MMI dosing increases in the blood. The last term, −*k*_8_*w*, represents the natural decay rate of TRAb.

The form of this model uses the idea of the functional size of the thyroid that was developed in our first article [26]. To be more precise, the rate of change of functional size (as opposed to thyroid size) over time was described as growth rate minus inactivation rate of functional size. The growth rate of functional size was modeled by a fraction of TSH concentration to the functional size when TSH is available in the blood serum. When TSH is not available, the growth rate decreases at physiologically standard constant rate *N*. By assumption (1), we replace TSH by TRAb (see Eq. (3)). The inactivation rate of functional size was influenced by the interaction of MMI concentration and the functional size [25].

### Stability analysis

We first analyze the stability of steady state for untreated Graves’ hyperthyroidism patients and subsequently analyze the stability of steady state for patients treated with MMI. The following remark describes the steady state for untreated patients:

#### **Remark 1**

When *s*(*t*)=0, we denote the hyperthyroid state as *E*_1_=(*x*_1_,*y*_1_,*z*_1_,*w*_1_) where

$$\begin{array}{*{20}l} {\kern100pt}x_{1} &= 0\\ y_{1} &=\frac{k_{3} k_{7}^{2}}{k_{4} k_{8} N \left(k_{8} k_{d}+k_{7}\right)}\\ z_{1}&= \frac{k_{7}}{k_{8} N}\\ w_{1} &=\frac{k_{7}}{k_{8}} \end{array} $$

#### **Theorem 1**

When *s*(*t*)=0 and *x*_0_=0, model (1)-(4) with all parameters positive has a unique steady state *E*_1_ (hyperthyroid state) in the hyperplane *x*=0, which is asymptotically stable.

#### *Proof*

By using Routh-Hurwitz criterion, we will prove that the hyperthyroid state is locally asymptotically stable inside the hyperplane *x*=0. We first assume *s*(*t*)=0 in the Eq. (1) and then consider the Jacobain matrix of this model, we obtain

$$J_{1}= \left[\begin{array}{cccc} \frac{-\left(k_{1}k_{a}z+k_{2}(x+k_{a})^{2}\right)}{(x+k_{a})^{2}} & 0 & \frac{-xk_{1}}{(x+k_{a})} & 0 \\ 0 &-k_{4} & \frac{wk_{3}}{w+k_{d}} & \frac{zk_{3}k_{d}}{(w+k_{d})^{2}}\\ -zk_{6} & 0 & \frac{-wk_{5}}{z^{2}}-xk_{6} & \frac{k_{5}}{z}\\ \frac{-k_{7}k_{b}}{(x+k_{b})^{2}} & 0 & 0 & -k_{8} \\ \end{array}\right ] $$

We next evaluate the Jacobian at the hyperthyroid state, *E*_1_=(*x*_1_,*y*_1_,*z*_1_,*w*_1_) and obtain:

$$J_{1}= \left[\begin{array}{cccc} -\frac{k_{1} k_{7}}{k_{8} N k_{a}}-k_{2} & 0 & 0 & 0 \\ 0 & -k_{4} & \frac{k_{3} k_{7}}{k_{8} k_{d}+k_{7}} & \frac{k_{3} k_{7} k_{8} k_{d}}{N \left(k_{8} k_{d}+k_{7}\right){}^{2}} \\ -\frac{k_{6} k_{7}}{k_{8} N} & 0 & -\frac{k_{5} k_{8} N^{2}}{k_{7}} & \frac{k_{5} k_{8} N}{k_{7}} \\ -\frac{k_{7}}{k_{b}} & 0 & 0 & -k_{8} \\ \end{array}\right] $$

The eigenvalues of Jacobian matrix can be found by solving the characteristic equation *det*(*J*_1_−*λI*)=0, which is *λ*^4^+*a*_1_*λ*^3^+*a*_2_*λ*^2^+*a*_3_*λ*+*a*_4_=0, where

$a_{1}=\frac {k_{1} k_{7}}{k_{8} N k_{a}}+\frac {k_{5} k_{8} N^{2}}{k_{7}}+k_{2}+k_{4}+k_{8} $

$a_{2}=\frac {k_{8} N k_{a} \left (k_{5} k_{8}^{2} N^{2}+\left (k_{2}+k_{4}\right) k_{8} \left (k_{5} N^{2}+k_{7}\right)+k_{2} k_{4} k_{7}\right)+k_{1} k_{7} \left (k_{5} k_{8} N^{2}+k_{7} \left (k_{4}+k_{8}\right)\right)}{k_{7} k_{8} N k_{a}}$

$a_{3}=\frac {k_{8} N k_{a} \left (\left (k_{2}+k_{4}\right) k_{5} k_{8} N^{2}+k_{2} k_{4} \left (k_{5} N^{2}+k_{7}\right)\right)+k_{1} k_{7} \left (k_{5} k_{8} N^{2}+k_{4} \left (k_{5} N^{2}+k_{7}\right)\right)}{k_{7} N k_{a}}$

$a_{4}=\frac {k_{4} k_{5} k_{8} N \left (k_{2} k_{8} N k_{a}+k_{1} k_{7}\right)}{k_{7} k_{a}}$

Since all parameters from the model are positive, *a*_1_>0,*a*_3_>0,*a*_4_>0. We will now verify the criteria: $a_{3}^{2}+a_{1}^{2}a_{4}-a_{1}a_{2}a_{3}<0$ to ensure the asymptotically stability of the hyperthyroid state inside the hyperplane *x*=0 [29].

$$\begin{aligned} a_{3}^{2}+a_{1}^{2}a_{4}-a_{1}a_{2}a_{3} &=-\frac{f}{k_{7}^{3} k_{8}^{2} N^{3} k_{a}^{3}} \\ &\quad<0 \end{aligned} $$

where

$$\begin{array}{*{20}l} f &=\left(k_{4}+k_{8}\right) \left(k_{5} N^{2}+k_{7}\right) \left(k_{5} k_{8} N^{2}+k_{4} k_{7}\right) \left(\left(k_{2}+k_{4}\right) k_{8} N k_{a}+k_{1} k_{7}\right) \\&\quad\left(k_{8} \left(k_{2}+k_{8}\right) N k_{a}+k_{1} k_{7}\right) \phantom{{}=} \left(k_{8} N k_{a} \left(k_{5} k_{8} N^{2}+k_{2} k_{7}\right)+k_{1} k_{7}^{2}\right) \\ & \quad>0 \end{array} $$

This completes the proof. □

#### **Remark 2**

We denote the euthyroid steady state as *E*_2_=(*x*_2_,*y*_2_,*z*_2_,*w*_2_). The analytical description of this state is not possible. So, we present the qualitative analysis of the existence of the euthyroid state and its stability.

#### **Theorem 2**

When *s*(*t*)=*c*>0 (treatment parameter) and *x*_0_>0, model (1)-(4) with all parameters positive has a unique euthyroid state *E*_2_=(*x*_2_,*y*_2_,*z*_2_,*w*_2_) in the positive orthant. Moreover, *z*_2_, *y*_2_, *w*_2_ are all decreasing functions of *c* if

$$\begin{array}{*{20}l} c < k_{1}z_{2}+k_{2}k_{a}+2k_{2}x_{2}- k_{1} x_{2}\left[\frac{k_{5}k_{7}k_{b}}{k_{8}(k_{b}+x_{2})^{2}}+k_{b}z_{2}^{2}\right]\frac{1}{(Nk_{5}+2k_{6}x_{2}z_{2})} \end{array} $$

#### *Proof*

We first assume *s*(*t*)=*c*>0 in the Eq. (1) where c is a positive real number or treatment parameter. To confirm the existence of euthyroid state in the positive orthant, we set the right hand side of (1)-(4) to zero;

5 $$\begin{array}{*{20}l} c-\frac{(k_{1}z)x}{k_{a}+x}-k_{2}x &=0  \end{array} $$

6 $$\begin{array}{*{20}l} \frac{(k_{3}z)w}{k_{d}+w}-k_{4}y &=0  \end{array} $$

7 $$\begin{array}{*{20}l} k_{5}\left(\frac{w}{z}-N\right)-k_{6}zx &=0  \end{array} $$

8 $$\begin{array}{*{20}l} k_{7}-\frac{k_{7}x}{k_{b}+x}-k_{8}w &=0  \end{array} $$

For euthyroid state *E*_2_=(*x*_2_,*y*_2_,*z*_2_,*w*_2_), we have an equation from (5)

9 $$\begin{array}{*{20}l} ck_{a}+(c-k_{1}z_{2}-k_{2}k_{a})x_{2}-k_{2}x_{2}^{2}=0  \end{array} $$

Equation (9) is the quadratic equation for *x*_2_, which has a positive root and a negative root for (*c*−*k*_1_*z*_2_−*k*_2_*k*_*a*_)<0 or (>0) with all parameters are positive. By Descarte’s Rule of Signs, we see there is a sign change for (9) so there exists a root *x*_2_ in the positive orthant.

From (8), we have a single equation

10 $$\begin{array}{*{20}l} w_{2}&=\frac{k_{7}k_{b}}{k_{8}(k_{b}+x_{2})}  \end{array} $$

Notice that if *x*_2_ lies in the positive orthant, then *w*_2_ lies in the positive orthant for all positive parameters. From (7), we have a quadratic equation for *z*_2_

11 $$\begin{array}{*{20}l} k_{6}z_{2}^{2}x_{2}+Nk_{5}z_{2}-k_{5}w_{2}=0  \end{array} $$

By Descarte’s Rule of Signs, we see there is a sign change for (11) for *x*_2_>0 and *w*_2_>0 so there exists a root *z*_2_ in the positive orthant. From (6), we have an equation

12 $$\begin{array}{*{20}l} y_{2}&=\frac{k_{3}z_{2}w_{2}}{k_{4}(k_{d}+w_{2})}  \end{array} $$

which also lies in the positive orthant for *z*_2_>0 and *w*_2_>0. Therefore, there exists a unique euthyroid state *E*_2_=(*x*_2_,*y*_2_,*z*_2_,*w*_2_) in the positive orthant when *c*>0.

Differentiating implicitly both sides of (9), (10),(11) and (12) with respect to c, we obtain the following partial differential equations:

13 $$\begin{array}{*{20}l} \frac{\partial x_{2}}{\partial c} &= \frac{k_{a}+\left(1-k_{1} \frac{\partial z_{2}}{\partial c}\right)x_{2}}{k_{1}z_{2}+k_{2}k_{a}+2k_{2}x_{2}-c}  \end{array} $$

14 $$\begin{array}{*{20}l} \frac{\partial w_{2}}{\partial c} &= \frac{-k_{7} k_{b}}{k_{8}(k_{b}+x_{2})^{2}} \frac{\partial x_{2}}{\partial c} \end{array} $$

15 $$\begin{array}{*{20}l} \frac{\partial z_{2}}{\partial c} &=\frac{-\partial x_{2}}{\partial c}\left[\frac{k_{5}k_{7}k_{b}}{k_{8}(k_{b}+x_{2})^{2}}+k_{b}z_{2}^{2}\right]\frac{1}{(Nk_{5}+2k_{6}x_{2}z_{2})}  \end{array} $$

16 $$\begin{array}{*{20}l} \frac{\partial y_{2}}{\partial c} &=\frac{k_{4}k_{d}k_{3}z_{2}\frac{\partial w_{2}}{\partial c} + \left(k_{4}k_{d}k_{3}w_{2}+k_{4}k_{3}w_{2}^{2}\right)\frac{\partial z_{2}}{\partial c}}{k_{4}(k_{d}+w_{2})^{2}} \end{array} $$

Substituting (15) into (13), we obtain an equation;

17 $$\begin{array}{*{20}l} \frac{\partial x_{2}}{\partial c} = \frac{k_{a}+x_{2}}{k_{1}z_{2}+k_{2}k_{a}+2k_{2}x_{2}-c- k_{1} x_{2}\left[\frac{k_{5}k_{7}k_{b}}{k_{8}(k_{b}+x_{2})^{2}}+k_{b}z_{2}^{2}\right]\frac{1}{(Nk_{5}+2k_{6}x_{2}z_{2})}}  \end{array} $$

Suppose the denominator of (17) is positive, we have $\frac {\partial x_{2}}{\partial c}$ greater than zero, then $\frac {\partial w_{2}}{\partial c}, \frac {\partial z_{2}}{\partial c}$ and $\frac {\partial y_{2}}{\partial c}$ are all negative, which implies *w*_2_, *z*_2_, *y*_2_ are all decreasing functions of *c*. □

#### **Theorem 3**

The euthyroid state *E*_2_=(*x*_2_,*y*_2_,*z*_2_,*w*_2_) is locally asymptotically stable in (1)-(4) when *s*(*t*)=*c*>0 and *w*=*Nz*.

#### *Proof*

Let $\overline {x}=x-x_{2}$, $\overline {y}=y-y_{2}$, $\overline {z}=z-z_{2}$ and $\overline {w}=w-w_{2}$. We define a Lyapunov function *V*:ℜ^4^→ℜ by the equation

18 $$\begin{array}{*{20}l} V(\overline{x},\overline{y},\overline{z},\overline{w})=\frac{\overline{x}^{2}}{2}+ \overline{x} \overline{w}+\frac{\overline{w^{2}}}{2} \end{array} $$

Without loss of generality, we see the system (1)-(4) becomes (19)-(22) in the new coordinates;

19 $$\begin{array}{*{20}l} \frac{d\overline{x}}{dt}&=-\frac{\overline{x}\overline{z}+\overline{x}z_{2}+x_{2}\overline{z}}{f_{1}+\overline{w}}-k_{2}\overline{x} \end{array} $$

20 $$\begin{array}{*{20}l} \frac{d\overline{y}}{dt}&=\frac{k_{3}\left(\overline{z}\overline{w}+z_{2}\overline{w}+w_{2}\overline{z} \right)}{f_{2}+\overline{w}} - k_{4} \overline{y}  \end{array} $$

21 $$\begin{array}{*{20}l} \frac{d\overline{z}}{dt}&=k_{5}\left(\frac{\overline{w}+w_{2}}{\overline{z}+z_{2}}-N \right)-k_{6}\overline{z}\overline{w}-k_{6} z_{2}\overline{w}-k_{6}\overline{z}w_{2}  \end{array} $$

22 $$\begin{array}{*{20}l} \frac{d\overline{w}}{dt}&=-\frac{k_{7}(x_{2}+\overline{x})}{f_{3}+\overline{x}}-k_{8}\overline{w}  \end{array} $$

For the system (19)-(22), (0,0,0,0) is an equilibrium point if *w*=*Nz*. Moreover *V*(0,0,0,0)=0 if $(\overline {x},\overline {y},\overline {z},\overline {w})=\mathbf {0}$, $V(\overline {x},\overline {y},\overline {z},\overline {w})>0$ for all $(\overline {x},\overline {y},\overline {z},\overline {w})$ in the positive orthant and

$$\begin{array}{*{20}l} \frac{dV}{dt}&=\frac{\partial V}{\partial \overline{x}}\frac{d\overline{x}}{dt}+\frac{\partial V}{\partial \overline{y}}\frac{d\overline{y}}{dt}+\frac{\partial V}{\partial \overline{z}}\frac{d\overline{z}}{dt}+\frac{\partial V}{\partial \overline{w}}\frac{d\overline{w}}{dt}\\ &=-(\overline{x}+\overline{w})\left(\frac{\overline{x}\overline{z}+\overline{x}z_{2}+x_{2}\overline{z}}{f_{1}+\overline{w}}+k_{2}\overline{x} +\frac{k_{7}(x_{2}+\overline{x})}{f_{3}+\overline{x}}+k_{8}\overline{w} \right)\\ &\quad<0 \end{array} $$

Therefore, the origin is locally asymptotically stable for (19)-(22) and thus euthyroid state is locally asymptotically stable for (1)-(4). This completes the proof. □

## Numerical simulations

### Data

We have 90 Graves’ hyperthyroidism patients data in which we have information for steady state levels of free thyroxine (FT4), free triiodothyronine (FT3), thyroid receptor stimulating antibodies (TRAb), thyroid peroxidase antibodies (TPOAb), thyroglobulin antibodies (TgAb), Methimazole (MMI) loading and maintenance dosage levels and age (Additional file 1). Serum TRAb is a bio-marker for Graves’ disease, however it is not a criterion for the dose of antithyroid drugs [11]. So, antithyroid drug (MMI) is prescribed based on solely FT4 and FT3 levels. When data was collected, FT4 and FT3 measured in SI units (pmol/L) that we converted FT4 to the conventional unit (in terms of pg/mL) by using the factor 0.7769 and converted FT3 to the conventional unit (in terms of ng/L) by using the factor 0.651 for our purposes [30]. Since FT4 data was collected from different labs across Sicily, Itlay, the normal reference range adopted by these labs was different. So, we have center the FT4 data for commonly used clinical reference range (7−18) pg/mL by using the formula $y=\frac {(18-7)*(x-LFT4)}{(UFT4-LFT4)}+7$, where *x* is a actual measurement of FT4 value (in pg/mL), *LFT*4 and *UFT*4 are lower and upper reference range values of FT4 used in the labs (in pg/mL). The same idea for the formula was used in our previous articles [26, 31]. For some patients in our data, TRAb was not measured in the first visit due to high levels of FT4 and that indicated the 1st evidence of hyperthyroidism. Just note that TSH were undetectable for many of our patients at the first clinical visit.

Based on the levels of FT4, MMI loading dosage started with six, four, three or two tablets per day for one or two months. Each tablet consists of 5 mg of MMI. After initial treatment, the loading dosage tablets were decreased for almost all patients by one tablet stepwise for every month. After loading dosage or during follow up, the maintenance dosage would be started when patients FT4 levels were controlled within normal range. Typically, one or half tablet per day was prescribed as the maintenance dosage and the entire MMI therapy lasts for about 12 to 24 months. Once patients achieved euthyroidism, MMI therapy would be stopped and patients were all asked to check their FT4 levels periodically to avoid relapses of hyperthyroidism. After stopping therapy, relapses may occur for some patients. If relapses occur, then MMI therapy would be administered again. In our data bank, we have some patients went through two courses of MMI treatment. Antibodies TRAb, TPOAb and TgAb are not measured routinely during therapy, as the healthcare system would not reimburse patients. A summary statistics of FT3, FT4, TRAb, TPOAb and TGAb for the 1st evidence of hyperthyroidism can be found in the Table 1.

**Table 1:** Summary statistics of FT3, FT4, TRAb, TPOAb and TGAb for 1st clinical visit of 90 patients

	FT3:	FT4:	TRAb:	TPOAb:	TGAb:
	(23 - 50) ng/L	(7 - 18) pg/mL	(0 - 1) U/mL	(< 100) U/mL	(< 100) U/mL
Mean	77.5	32.6	16.05	793.5	795.2
Median	70.9	31.2	11.4	263.5	138.5
Mean variability	77.5±37.5	32.6±11.3	16.05±22.5	793.5±1822.2	795.2±3760

### Initial conditions

We can calculate the initial concentration of the Methimazole (MMI) in the blood serum as follows

$$\begin{array}{*{20}l} x_{0}=\frac{\text{Loading dose}}{\text{Volume of distribution in blood serum}} \end{array} $$

Loading dose information can be obtained from data and can assume the volume of distribution in blood serum approximately 3 L. The initial state of the FT4 (*y*_0_) and TRAb (*w*_0_) can be measured at the steady state levels for each patient so it can be found from the patient’s data. The initial state of the functional size of the thyroid gland may be calculated via the equilibrium argument from Eq. (1), if the parameters *k*_1_, *k*_*a*_ and dose function *s*(*t*) are known; that is,

$$\begin{array}{*{20}l} z_{0}&=\frac{(s(t)-k_{2}x_{0})(k_{a}+x_{0})}{k_{1}x_{0}} \end{array} $$

We can calculate *s*(*t*) and *k*_2_ from MMI dosing information but parameters *k*_1_ and *k*_*a*_ are difficult to estimate due to high variability in patients’ thyroid gland MMI uptake and MMI distribution in blood and body volume. So, we consider the functional size as an hidden compartment and assume *z*_0_=30 mL for hyperthyroidism patients. With this assumption, we estimate parameter values *k*_1_ and *k*_*a*_ in next section. Therefore, our initial conditions for numerical simulation is of the form *E*_0_=(*x*_0_,*y*_0_,30,*w*_0_).

### Parameter estimates

With twelve positive parameters and a dose (rate) function *s*(*t*) in this model, we have to determine the numerical values for these parameters and a function via patients data, thyroid literature and equilibrium argument of hyperthyroidism state. A summary of the parameter estimates can be found in Table 2. In Eq. (1), we first calculate the dose function *s*(*t*)=*c* based on the loading dosage schedule information and bio-availability of MMI (*f*=93*%*). Typically, dosing schedule of a patient is obtained from the data. Suppose the loading dosing schedule is 30 mg/day for 45 days for the average man with body volume 59.71 L, then

$$s(t)= \left\{\begin{array}{ll} \frac{0.93 \times 30~\text{mg/day} \times 45\, \text{days}}{59.71\mathrm{L}} = 21.027\, \text{mg/L} &\quad \text {if} \quad 0 \leq t \leq 45 \\ 0 & \quad \text {Otherwise} \end{array}\right. $$

The maximum uptake rate of MMI is obtained from the information in literature [28]. Huang G et. al observed that as MMI dosage varies from 5 to 15 mg/day, intrathyroid levels of MMI increased with increasing dose, but without significant increase when 15 mg/day. Their results indicate the maximum saturation rate of intrathyroidal concentration of MMI 15 mg/day. Suppose the normal functional size of the gland in hyperthyroidism is *z*_0_=30 mL, then we can estimate $k_{1}=\frac {15}{30*59.71}=8.374 \times 10^{-3}~ \text {mg/mL*L day}$. Next, the literature says MMI excretion happens in about 12 of the day for the maximum amount of 15 mg/day. With this information, we determined the value of *k*_*a*_=0.358068 mg/L through simulations. The rate of elimination of MMI through non-specific mechanism is assumed to be exponential which can be calculated as $k_{2}=\frac {ln(2)}{5 \,\text {h}}=3.3271 \text {1/day}$.

**Table 2:** Parameter Estimates, Description, Units and Source

Parameter	Description	Value	Source	Units
*k*_1_	Relative maximum uptake rate of MMI	8.374×10^−3^	Literature [28]	*mg*/(*mL*∗*Lday*)
*k*_2_	Elimination rate of MMI	3.3271	Estimated	1/*day*
*k*_*a*_	Michealis-Menten constant for half maximal uptake rate of MMI	0.358068	Simulation	*mg*/*L*
*k*_3_	Relative maximum secretion rate of FT4	0.119	Calculation	*pg*/(*mL*^2^*day*)
*k*_*d*_	Michealis-Menten constant for half maximal secretion rate of FT4	0.05	Simulation	*U*/*mL*
*k*_4_	Elimination rate of FT4	0.099021	Estimated	1/*day*
*k*_5_	Relative growth rate of the functional size of thyroid gland	1×10^6^	Simulation	*mL*^3^/(*U*∗*day*)
*N*	Maximal growth ratio	0.833	Calculation	*U*/(*mL*^2^)
*k*_6_	Thyroid inactivation rate constant	0.001	Simulation	*mL*/*mg*∗*day*
*k*_7_	Maximal production rate of TRAb	0.875	Calculation	*U*/(*mL*∗*day*)
*k*_*b*_	Inhibition rate of TRAb	1.5	Simulation	*mg*/*L*
*k*_8_	Elimination rate of TRAb	0.035	Estimated	1/*day*

In Eq. (2), we first estimate *k*_*d*_ through **fmincon** optimization procedure in Matlab R2015a with lower bound 0.05 and upper bound 0.1. Next, we calculate *k*_3_ via the equilibrium argument with initial steady state, *E*_0_=(*x*_0_,*y*_0_,*z*_0_,*w*_0_). Note when *x*_0_=0, the initial steady state represent hyperthyroidism state. From the data, we have information for hyperthyroidism state for each patient from their 1st visit, so the initial concentration of TRAb (*w*_0_), initial concentration of FT4 (*y*_0_) and the functional size of thyroid gland (*z*_0_) yields;

$$\begin{array}{*{20}l} \frac{dy}{dt}&=0=\frac{k_{3}z_{0}w_{0}}{(k_{d}+w_{0})}-k_{4}y_{0}\\ k_{3}&=\frac{k_{4}y_{0}(k_{d}+w_{0})}{z_{0}w_{0}} \end{array} $$

The parameter *k*_4_ is the excretion rate of FT4 which assumed to be exponential due to a non-specific mechanism so it can be calculated as $k_{4}=\frac {ln(2)}{7 \text {days}}=0.099021~\text {1/day}$.

In Eq. (3), we determine the value of *N* from the initial concentration of TRAb (*w*_0_) and the functional size of the thyroid gland (*z*_0_) at the hyperthyroid state or from the patients data. Again, the equilibrium argument was used to estimate the parameter $N=\frac {w_{0}}{z_{0}}$ when the MMI treatment was not given. The parameters *k*_5_ and *k*_6_ were assumed to be one as found through the simulation.

In Eq. (4), we determine the maximal production rate of TRAb at the hyperthyroid state of patient. More precisely, the parameter estimate of *k*_7_=*k*_8_*w*_0_ in the absence of MMI treatment. The parameter *k*_8_ was the death rate of TRAb, which we assume to be exponential in the model and that was calculated as $k_{8}=\frac {ln(2)}{20~\text {days}}=0.035~ \text {1/day}$. The parameter *k*_*b*_ was the half-maximal inhibition concentration of TRAb (Michalies-Menten constant) that can be estimated with **fmincon** procedure with lower bound 3 and upper bound 12.

### Simulation of the model

**Fig. 3:**
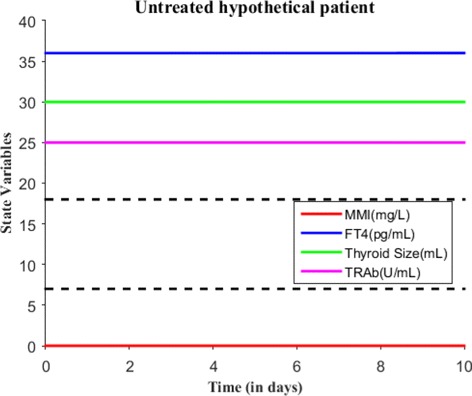
With *E*_0_=(0,36,30,25) and *s*(*t*)=0 mg/L/day, the untreated patient steady state levels remains same for 10 days

**Fig. 4:**
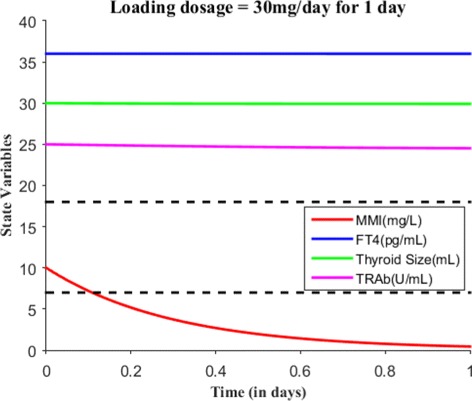
With *E*_0_=(10,36,30,25), suppose the loading dosage = 30 mg is given for 1 day which results in almost no change in patient’s FT4 steady state levels. The dotted lines are shown to indicate the lower and upper reference limit of FT4

**Fig. 5:**
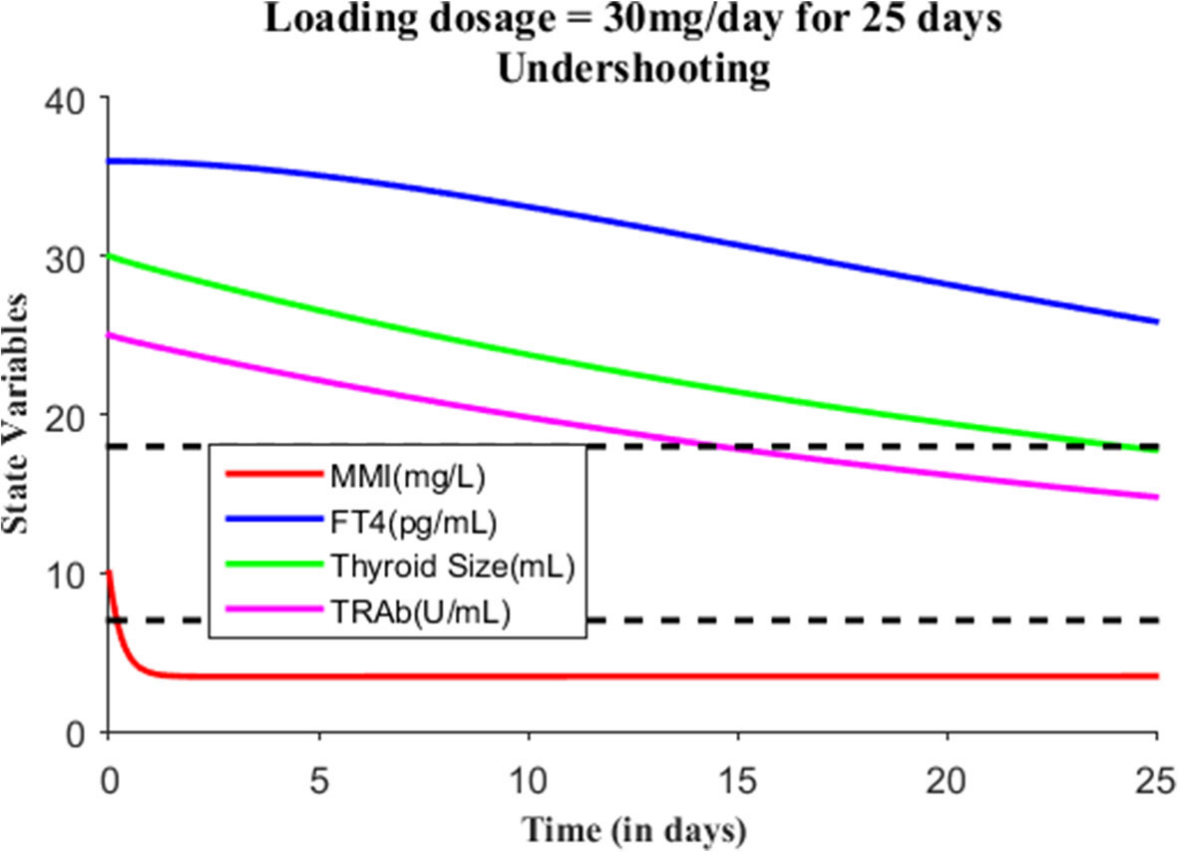
With *E*_0_=(10,36,30,25), suppose the loading dosage = 30 mg/day is given for 25 days which results in subclinical hyperthyroidism (Undershooting)

**Fig. 6:**
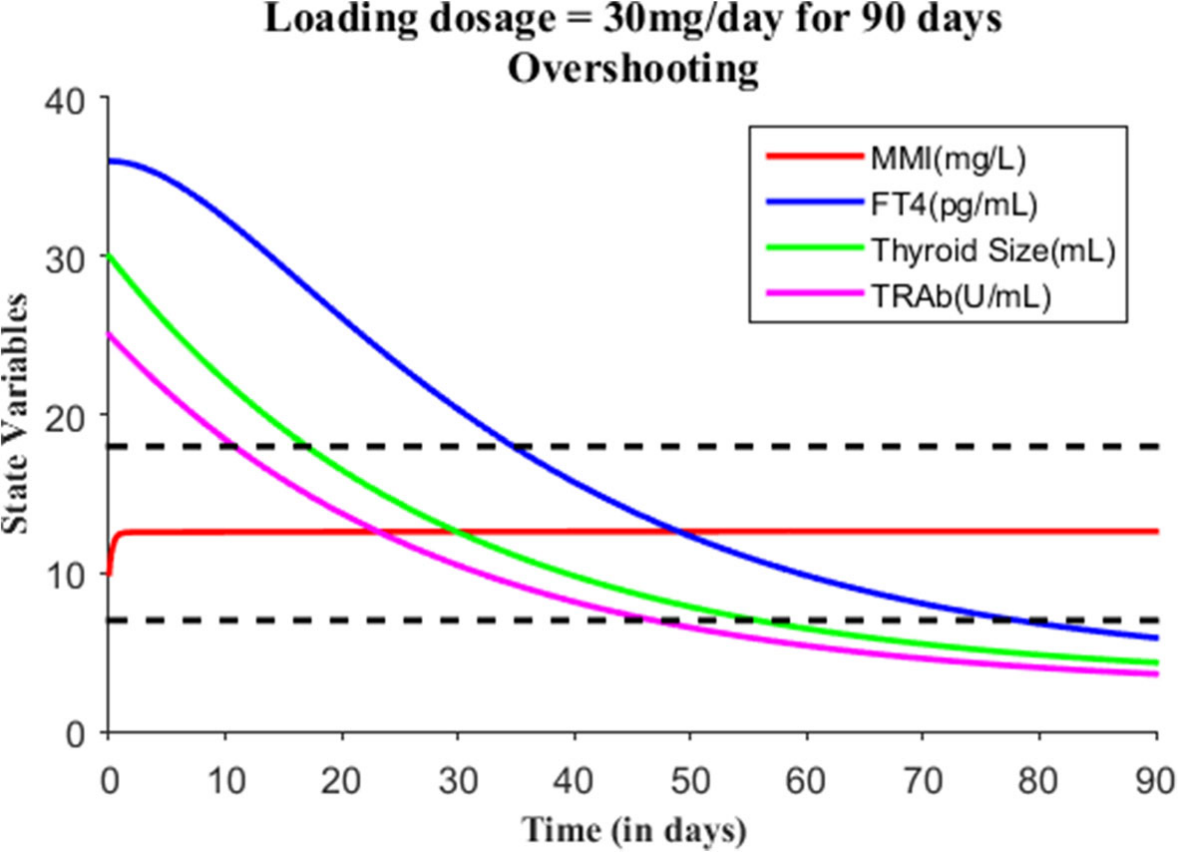
With *E*_0_=(10,36,30,25), suppose the loading dosage = 30 mg/day is given for 90 days which results in hypothyroidism (overshooting)

We will now numerically simulate our model for a finite time period based on the MMI treatment. Consider a hypothetical hyperthyroid patient whose initial state is *E*_0_=(0,36,30,25) and parameter values are taken from Table 2. Without MMI treatment (i.e., *s*(*t*)=0), Fig. 3 shows the patient steady state levels remains the same for 10 days. We will now discuss various treatment scenarios with the assumption of body volume of this patient 59.71L. Suppose when effective dose of 30 mg of MMI is given for 1 day, the initial state becomes *E*_0_=(10,36,30,25) and Fig. 4 shows the patient has almost no change in their dynamics. Just recall we assumed one single large MMI dose is taken everyday, which is absorbed immediately and completely by the patient’s body. So, the initial state was the last point in the previous run or simulation. Suppose the effective loading dosage of 30 mg/day are administered for 25 days, Fig. 5 shows the patient’s FT4 levels decreased from 36 pg/mL but ended up in the subclinical hyperthyroidism (Undershooting). Suppose the effective loading dosage schedule is maintained as 30 mg/day for 90 days, Fig. 6 shows the patient’s FT4 levels decreased below the lower reference range of FT4 which in turn resulted in hypothyroidism (overshooting). So, in order to avoid overshooting, the loading dosage should be administered less than 90 days.

**Fig. 7:**
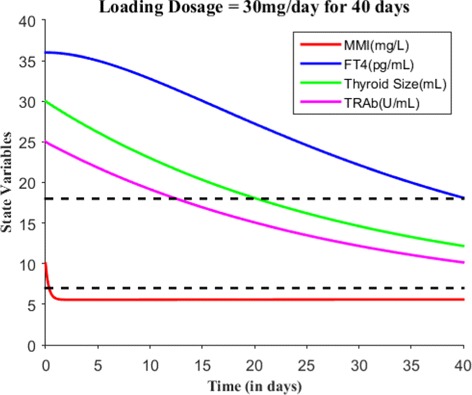
With *E*_0_=(10,36,30,25), suppose the loading dosage = 30 mg/day is given for 40 days which results in patient’s FT4 levels close to the upper normal reference limit

**Fig. 8:**
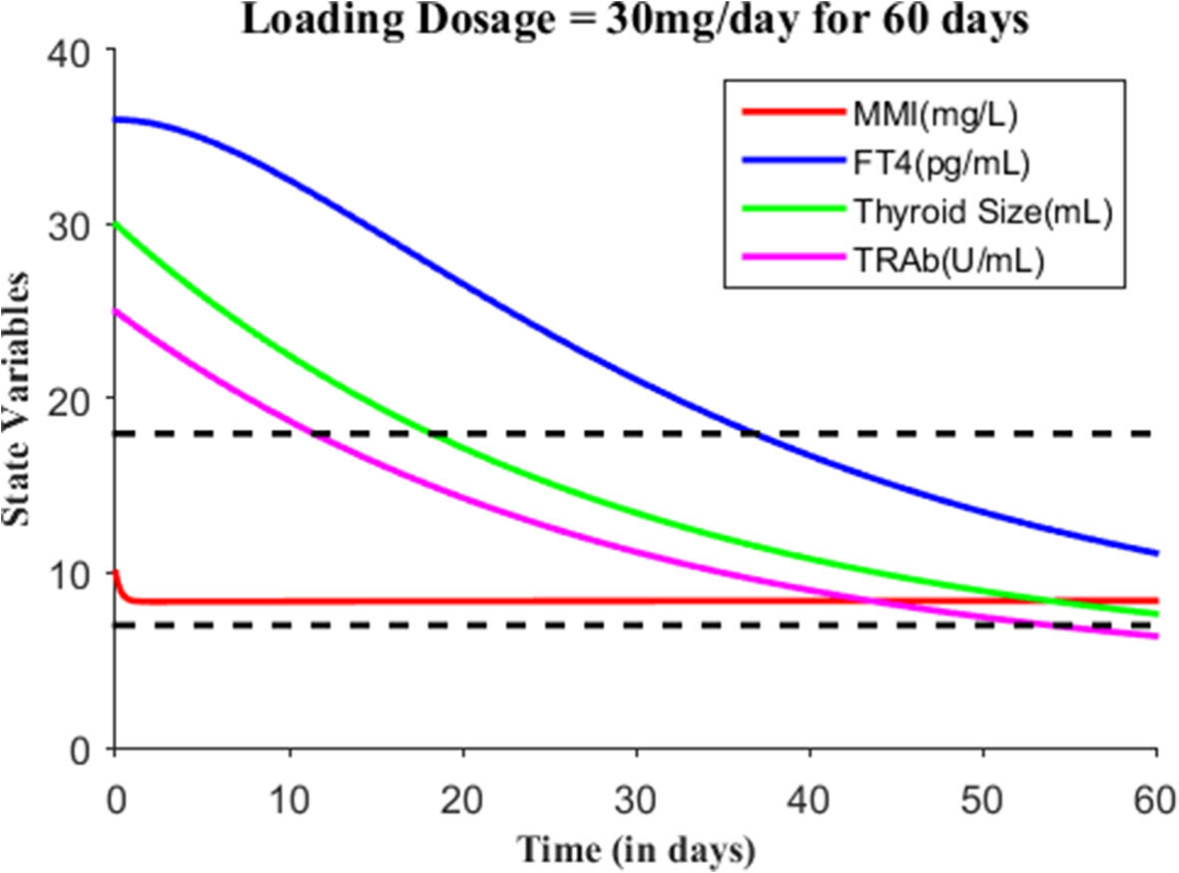
With *E*_0_=(10,36,30,25), suppose the loading dosage = 30 mg/day is given for 60 days which results in patient’s FT4 levels within the normal reference range

**Fig. 9:**
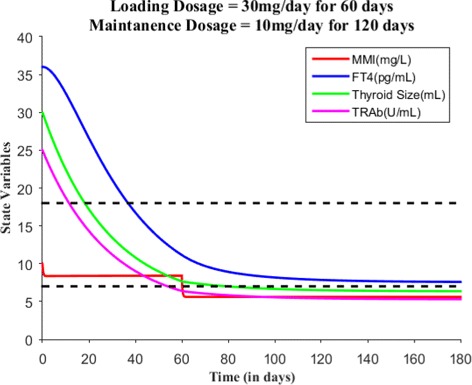
With *E*_0_=(10,36,30,25) and the loading dosage = 30 mg/day for 60 days results in patient’s FT4 levels within normal reference limit. After that, the maintenance dosage = 10 mg/day is given for 120 days in order to control FT4 levels within normal reference range

**Fig. 10:**
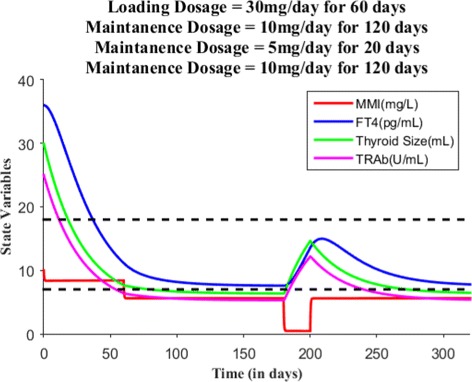
After the oral administration of loading dosage = 30 mg/day (for 60 days) and maintenance dosage =10 mg/day (for 120 days), patient’s FT4 levels reaches closer to lower normal reference limit, so MMI dosage is lowered to 5 mg/day for 20 days. This in turn increased the levels of FT4 and then maintenance dosage continued for another 120 days

**Fig. 11:**
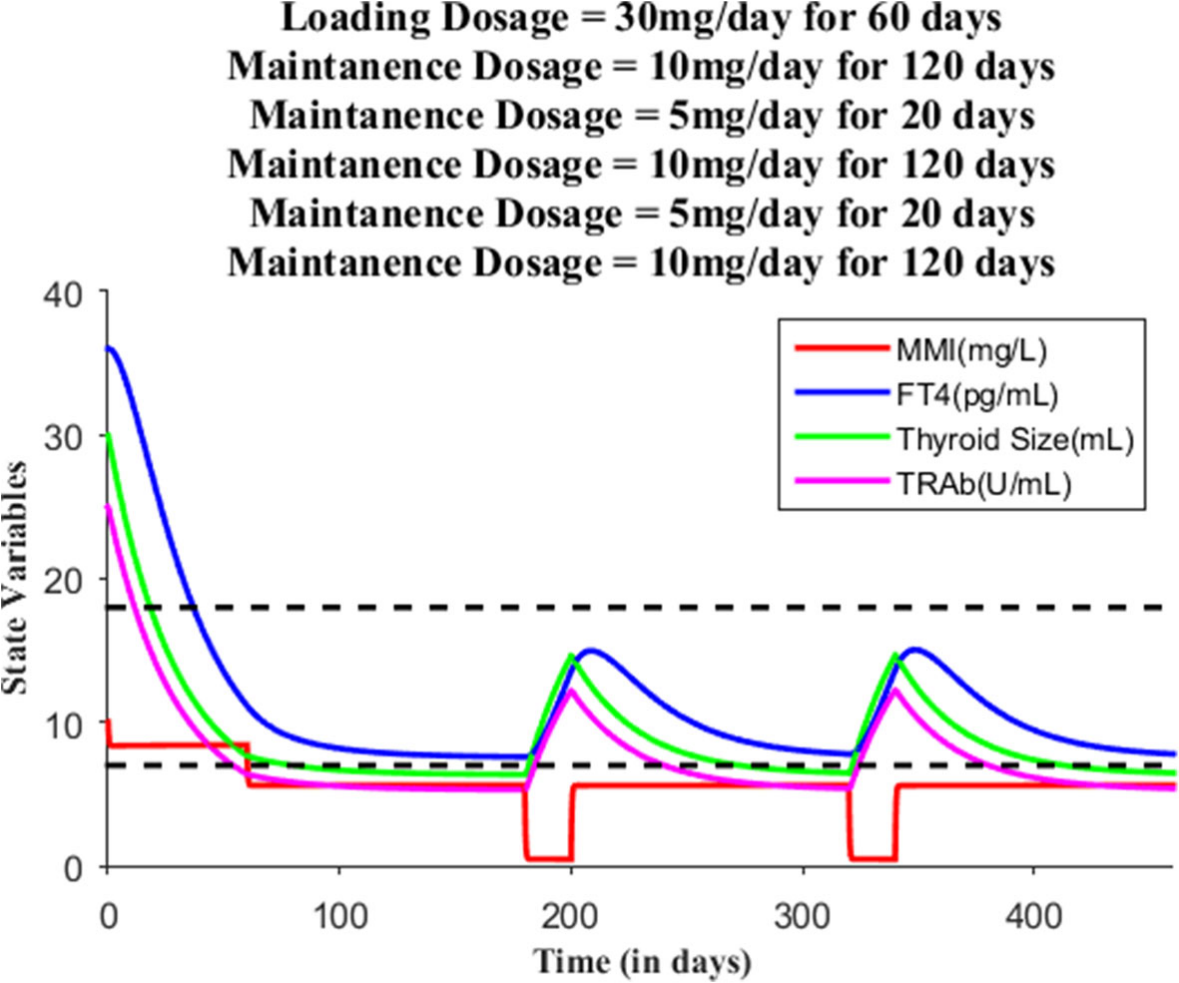
When patient’s FT4 levels reaches again closer to the lower normal reference limit, the maintenance dosage is lowered to 5 mg/day from 10 mg/day and then the regular maintenance dosage continued for up to 120 days in order to maintain FT4 levels within normal reference limit

**Fig. 12:**
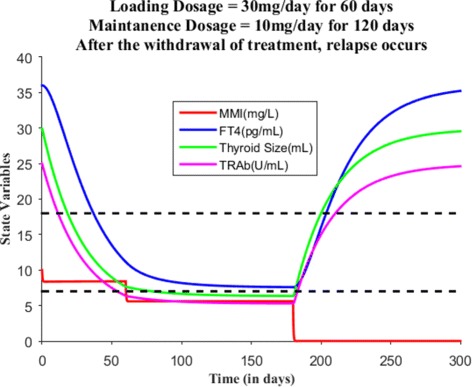
After the withdrawal of MMI treatment, the relapse of hyperthyroidism had occurred for the hypothetical patient

Suppose the loading dose is given 30 mg/day for 45 days, Fig. 7 shows patient’s FT4 levels decreased closer to the upper normal reference limit. Suppose the loading dose is given 30 mg/day for 60 days, Fig. 8 shows patient’s FT4 levels decreased to the normal reference range. Next, our goal is to maintain the FT4 levels within the reference range, so the loading dose of MMI is reduced to 10 mg/day and given for 120 days. Figure 9 shows the effect of MMI loading and maintenance dosage schedule for 180 days. After 180 days of treatment, patient’s FT4 levels are closer to lower normal reference limit. So, MMI dosage levels further lowered to 5 mg/day for 20 days which in turn increased FT4 levels within the normal reference range and then the regular maintenance dose of 10 mg/day is given for another 120 days (see Fig. 10). This cycle of treatment is repeated again for another 140 days (see Fig. 11) and the patient’s FT4 levels are completely controlled within the reference range. Relapse of hyperthyroidism is a tricky mechanism after stopping the MMI treatment, which may or may not occur for patients. If relapse occurs, then the model can explain this mechanism. Suppose if we assume the treatment of patient is stopped after one loading and maintenance dosing schedule (that is after 180 days), see Fig. 12 that shows the relapse of hyperthyroidism.

## Model validation

In this section, we will validate our model, so our task now is to simulate the model numerically for finite time and compare it with time course data. For validation purposes, we will arbitrarily choose four patients data from our data bank and calculate patient-specific and medication parameters using their measurement data, which in turn will be fed into the model for finite time simulation. See Table 3 for these four patients parameter values. The simulation of the model will be done in accordance with patient’s MMI loading and maintenance dosing schedule. The dosing schedule of these patients will also help us calculate the dosing rate function *s*(*t*)=*c*. So, we fix all parameters during simulation except the dosing rate function *c*. To analyze the simulation output, we have estimated a root mean square error reference interval for FT4 and TRAb from patients’ data. To estimate the reference interval, we eliminated patients with missing TRAb data. In other words, we considered only patients with at least three TRAb values at different time-points in the data set (27 patients). So, we have obtained a non-normalized reference interval (0.88, 36.40) for FT4 and (0.17, 15.73) for TRAb respectively (Additional file 2).

**Table 3:** Individual patient parameter values

Parameter	Patient# 20	Patient# 31	Patient# 55	Patient# 70
*k*_1_	8.374×10^−3^	8.374×10^−3^	8.374×10^−3^	8.374×10^−3^
*k*_2_	3.3271	3.3271	3.3271	3.3271
*k*_*a*_	0.358068	0.358068	0.358068	0.358068
*k*_3_	0.085	0.08975	0.08992	0.11784
*k*_*d*_	0.067	0.07	0.081	0.075
*k*_4_	0.099021	0.099021	0.099021	0.099021
*k*_5_	1×10^6^	1×10^6^	1×10^6^	1×10^6^
*N*	0.250	0.058	0.207	0.293
*k*_6_	0.001	0.001	0.001	0.001
*k*_7_	0.26	0.061	0.22	0.308
*k*_*b*_	4.95	11.8	4.09	3.15
*k*_8_	0.035	0.035	0.035	0.035

Note: The values of *k*_3_, *k*_*d*_, *N*, *k*_7_ and *k*_*b*_ varies among patients

We will first explain patient *#*20 time course data and then simulate the model in accordance with MMI treatment. In November 2010, this patient was diagnosed with Graves’ hyperthyroidism. At the first clinical visit, patient’s FT4 and TRAb levels were 25.63 pg/mL and 7.5 U/mL respectively with respect to normal reference ranges of FT4, (7 - 18) pg/mL and TRAb, (0 - 1) U/mL. At this visit, this patient was put under MMI treatment. A loading dosage was started with 6 tablets/day and asked to decrease tablets consumption by one stepwise every month. So, patient *#*20 took 6 tablets/day (i.e., 30 mg/day) for first month, 5 tablets/day (i.e., 25 mg/day) for second month and 4 tablets/day (i.e., 20 mg/day) for third month. After third month, patient followed up for check-up and the dosage was lowered to 2 tablets/day (i.e., 10 mg/day) for next three months because FT4 levels were 17.7 pg/mL (close to upper normal reference limit) and TRAb levels were not measured at this visit. After six months, patient visited the clinic and FT4 levels were measured 13.8 pg/mL (but not TRAb) and the dosage was further lowered to 1 tablet/day and prescribed for another six months. Now, after twelve months or 1 year, patient’s FT4 and TRAb was 13.14 pg/mL and 0.7 U/mL so the dosage level continued with same amount 1 tablet/day for another six months. Finally, after 1 year and six months, FT4 and TRAb was 12.46 pg/mL and 0.6 U/mL; therefore MMI dosage was stopped.

**Fig. 13:**
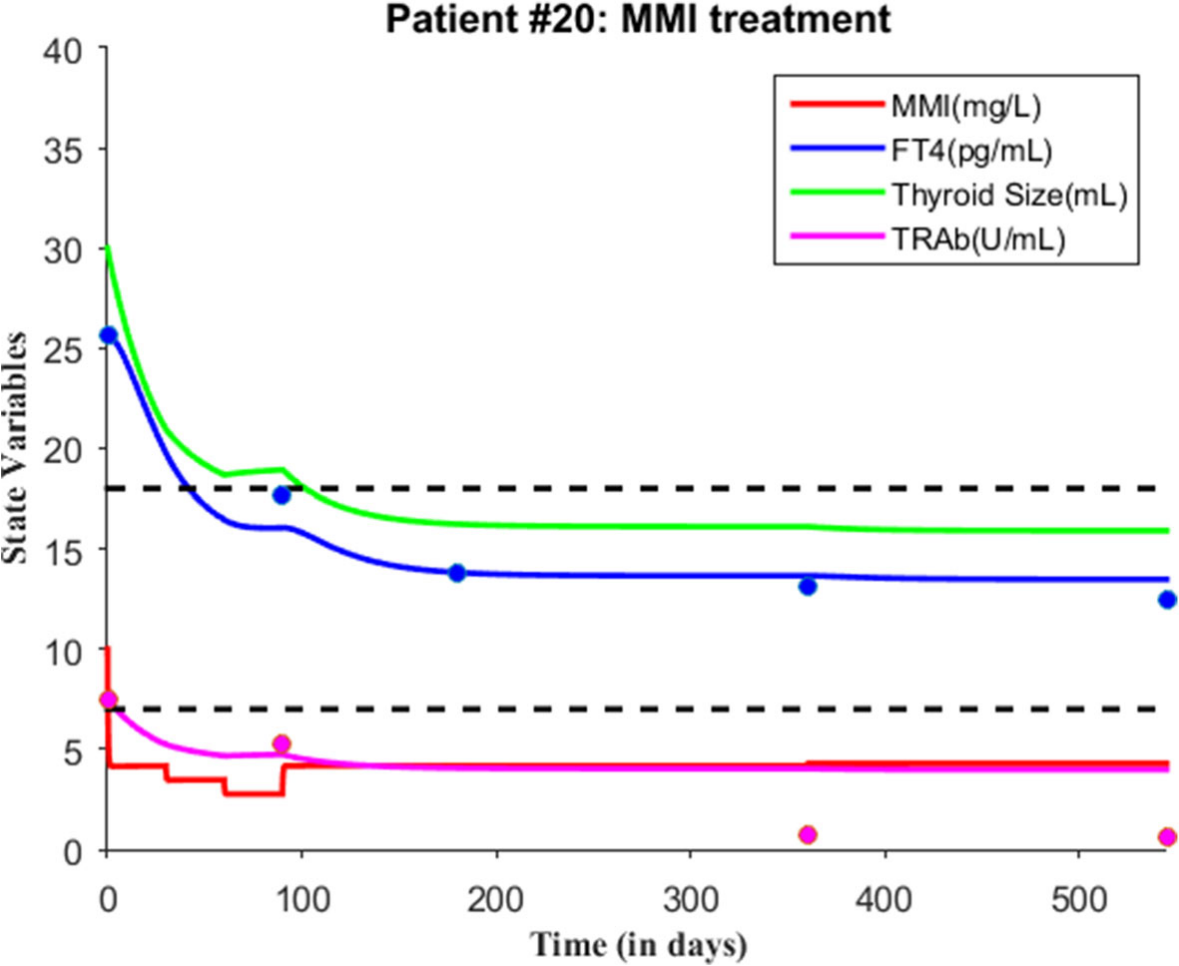
A course of MMI treatment is given for patient #20. Both FT4 and TRAb data is plotted with the simulated solutions. The root mean square error for the fit of FT4 and TRAb data is 0.889 and 2.388 respectively. Blue dots indicate FT4 levels while magenta dots indicate TRAb levels in the figure

Next, we simulate the model for patient #20 based on MMI loading and maintenance dosage information. Depending upon the consumption of amount of MMI, we calculated the parameter value *c* for each time period and run the model. For the 1st time period (1 month), the value of *c*=14.02 and the initial value *x*_0_=30/3=10 mg/L, *y*_0_=25.63 pg/mL, *z*_0_=30 mL and *w*_0_=7.5 U/mL. For the 2nd time period (1 month), the value of *c*=12.07 and the initial value was the last point in the previous run or simulation. For the 3rd time period (1 month), the value of *c*=9.6534 and the initial value was the last point in the previous run. For the 4th time period (3 months), the value of *c*=13.8663 and the initial value was the last point in the previous simulation. For the 5th and 6th time period (i.e., 1 year), the value of *c*=14.173 and the initial value was the last point in the previous run. The other parameter values were all fixed in the simulation (see Table 3 and Fig. 13). To find the accuracy of model fit to data, we calculate a measure of prediction; the root mean square error. It is computed from the square root of the average sum of squared residuals of the fit. For patient #20 data, we found the root mean square error for the fit of FT4 and TRAb data is 0.889 and 2.388 respectively. It is probably a good measure of accuracy for the available time course data, so generating patient treatment schedule may be reliable for patient #20.

As before, we first explain patient #31 time course data and then proceed with model validation. At the first visit, this patient’s FT4 and TRAb levels were 26.43 pg/mL and 1.74 U/mL respectively, which indicates Graves’ hyperthyroidism in accordance with normal reference range. So, this patient was put under MMI treatment. The treatment initiated with loading dose of 4 tablets/day for 1st month, lowered stepwise to 1 tablet/day. That is, 3 tablets/day for 2nd month, 2 tablets/day for 3rd month and 1 tablet/day for 4th month. After 4 months taking MMI, patient’s follow-up revealed FT4 levels were 20 pg/mL and TRAb was not measured at that time. The treatment was continued with 2 tablets/day for 1 month and then 1 tablet/day for 23 months. In a subsequent follow-up, MMI treatment was discontinued because FT4 and TRAb levels were 11.5 pg/mL and 0.45 U/mL respectively. Patient #31 followed-up for check up after 2 years of no treatment, FT4 and TRAb levels were 11.36 pg/mL and 0.43 U/mL normal.

**Fig. 14:**
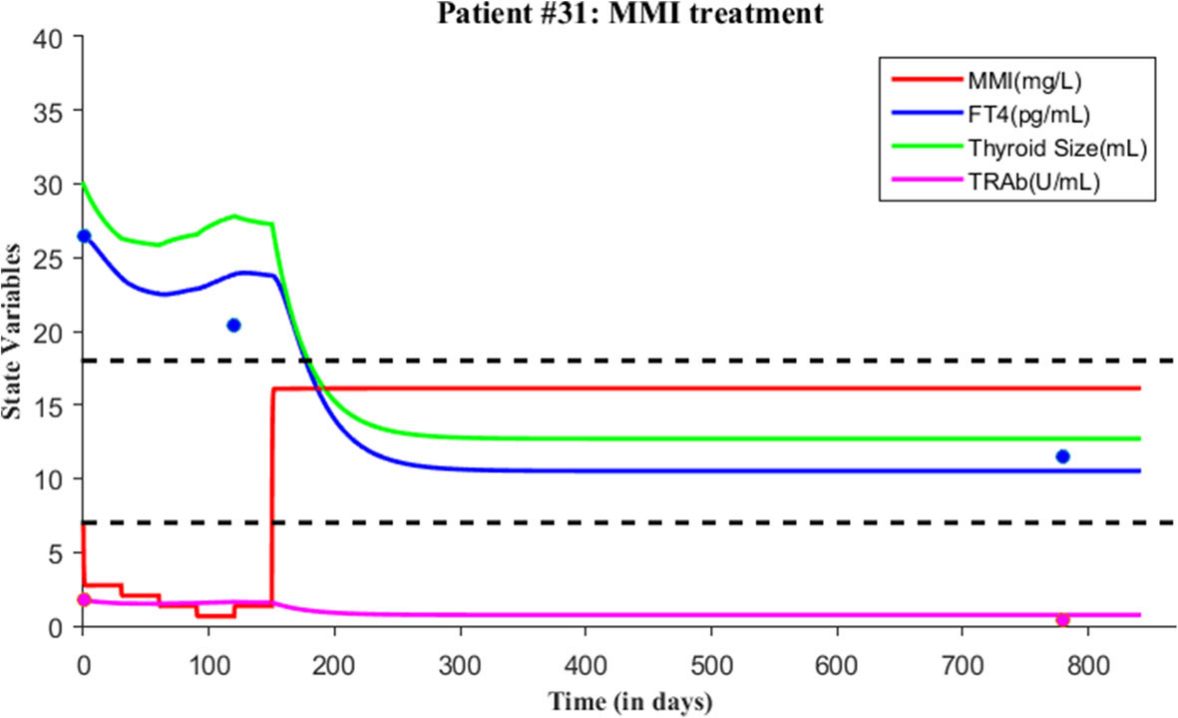
A course of MMI treatment is given for patient #31. Both FT4 and TRAb data is plotted with the simulated solutions. The root mean square error for the fit of FT4 and TRAb data is 1.9892 and 0.2303 respectively. Blue dots indicate FT4 levels while magenta dots indicate TRAb levels in the figure

Next, we simulate the model for patient #31 based on MMI loading and maintenance dosage information. Based on dosages, we calculate the value of c. For the 1st time period (i.e., 1 month), the value of *c*=9.3456 and the initial value *x*_0_=20/3=6.67 mg/L, *y*_0_=26.43 pg/mL, *z*_0_=30 mL and *w*_0_=1.74 U/mL. For the 2nd time period (i.e., 1 month), the value of *c*=7.01 and the initial value was the last point in the previous run. For the 3rd time period (i.e., 1 month), the value of *c*=4.6726 and the initial value was the last point in the previous run. For the 4th time period (i.e., 1 month), the value of *c*=2.3363 and the initial value was the last point in the previous simulation. For the 5th time period (i.e., 1 month), the value of *c*=4.6726 and the initial value was the last point in the previous simulation. For the 6th time period (i.e., 23 months), the value of *c*=53.7345 and the initial value was the last point in the previous run. The total treatment time period was about 840 days (see Fig. 14 and Table 3). There is a lack of data for patient #31, however we fit the model to the available data and provide a measure of prediction; the root mean square error (RMSE). The RMSE for the fit of FT4 and TRAb data is 1.9892 and 0.2303 respectively. The smaller RMSE for TRAb and slightly larger RMSE for FT4 indicates a reasonable amount of accuracy so generating patient treatment schedule may be reliable for patient #31.

As before, we first explain patient #70 time course data and then validate the model. At the first clinical visit, this patient’s FT4 and TRAb levels were 35.04 pg/mL and 8.8 U/mL respectively, which indicates Graves’ hyperthyroidism in accordance with normal reference range. So, this patient was put under MMI treatment at this visit. MMI dosage was prescribed as 6 tablets/day for first month, 5 tablets/day for second month, 4 tablets/day for third month, 3 tablets/day for fourth month, 2 tablets/day for fifth month and 1 tablet/day from sixth month to tenth month (that is to say four months of maintenance dosage). After tenth month, patient’s FT4 and TRAb were 9.3 pg/mL and 1.3 U/mL, so MMI treatment was continued with 1 tablet/day for another seven months. So, after seventeen months, patient followed up for check-up and now FT4 and TRAb were 8.97 pg/mL and 1.4 U/mL. Since FT4 measurement was closer to the lower reference range, MMI treatment was stopped for six months after this visit but asked to follow up on the thyroid status. Then, after six months, patient’s FT4 and TRAb levels were 31.2 pg/mL and 4.7 U/mL respectively so relapse occurred. A second course of MMI treatment started again with 4 tablets/day for 1 month, 2 tablets/day for three months and then 1 tablet/day for three months. In the follow-up of after seven months, patient’s FT4 and TRAb levels were 9.87 pg/mL and 0.9 U/mL respectively. MMI treatment continued with lower dosage 1 tablet/day for another six months and then patient FT4 levels remained as 9.87 pg/mL but TRAb levels decreased to 0.3 U/mL. Finally, treatment was stopped.

**Fig. 15:**
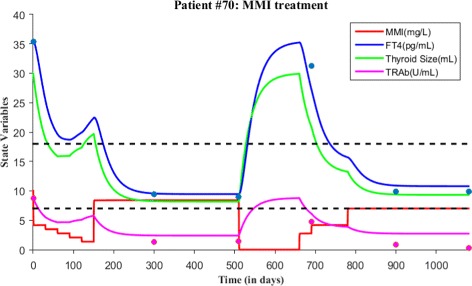
Two courses of MMI treatment is given for patient #70. Both FT4 and TRAb data is plotted with the simulated solutions. The root mean square error for the fit of FT4 and TRAb data is 1.7006 and 1.5084 respectively. Blue dots indicate FT4 levels while magenta dots indicate TRAb levels in the figure

We now simulate the model for patient #70 based on MMI loading and maintenance dosage information. For the 1st time period (i.e., 1 month), the value of *c*=14.02 and the initial value *x*_0_=30/3=10 mg/L, *y*_0_=35.04 pg/mL, *z*_0_=30 mL and *w*_0_=8.8 U/mL. For the 2nd time period (i.e., 1 month), the value of *c*=11.6817 and the initial value was the last point in the previous run or simulation. For the 3rd time period (i.e., 1 month), the value of *c*=9.3456 and the initial value was the last point in the previous run. For the 4th time period (i.e., 1 month), the value of *c*=7.0089 and the initial value was the last point in the previous simulation. For the 5th time period (i.e., 1 month), the value of *c*=4.6726 and the initial value was the last point in the previous simulation. For the 6th time period (i.e., 11 months), the value of *c*=25.6996 and the initial value was the last point in the previous run. After 17 months, treatment was stopped so we set the value of *c*=0.001 and run the model for six months. Relapse of hyperthyroidism occurred so the second course of MMI treatment started. For the 1st time period(i.e., 1 month), the 2nd time period (i.e., 3 months) and 3rd time period (i.e.,10 months), the values of *c* are 9.3456,14.02 and 23.3625 respectively. All parameter values are fixed in the simulation except *c* (see Table 3 and Fig. 15). To determine the accuracy of model fit to data, we calculated the root mean square error (RMSE) for the model fit of FT4 and TRAb data, which is 1.7006 and 1.5084 respectively. The smaller RMSE indicates a reasonable amount of accuracy so generating patient treatment schedule may be reliable for patient #70.

As before, we first explain patient #55 time course data and proceed to model validation. At the first clinical visit, this patient’s FT4 and TRAb levels were 27.01 pg/mL and 6.22 U/mL respectively, which indicates Graves’ hyperthyroidism in accordance with normal reference range. So, this patient was put under MMI treatment at this visit. MMI dosage was prescribed as 3 tablets/day for first month, 2 tablets/day for second month and 1 tablets/day for third month. After third month during follow-up, patient’s FT4 levels measured as 10.03 pg/mL and TRAb not measured, so MMI treatment was continued with 1 tablet/day for another six months. After nine months, patient’s FT4 and TRAb levels were 8.99 pg/mL and 1.17 U/mL respectively. Since FT4 measurement was closer to the low normal reference limit, MMI treatment was stopped. During follow-up after 23 months of no treatment, patient’s FT4 and TRAb levels were 40.28 pg/mL and 2.46 U/mL respectively and therefore relapse occurred. A second course of MMI treatment started with loading dose 4 tablets/day for first month, 3 tablets/day for second month and 2 tablets/day for third month. After third month during follow-up, patient’s FT4 levels were 12.41 pg/mL and TRAb levels not measured. Treatment was continued with lower dosage 1 tablet/day for another five months and then FT4 levels were measured as 10.79 pg/mL and TRAb not measured. Treatment continued again for another 11 months with lower dosage of 1 tablet/day. In a subsequent follow-up, patient’s FT4 levels and TRAb levels were 11.36 pg/mL and 0.7 U/mL respectively.

**Fig. 16:**
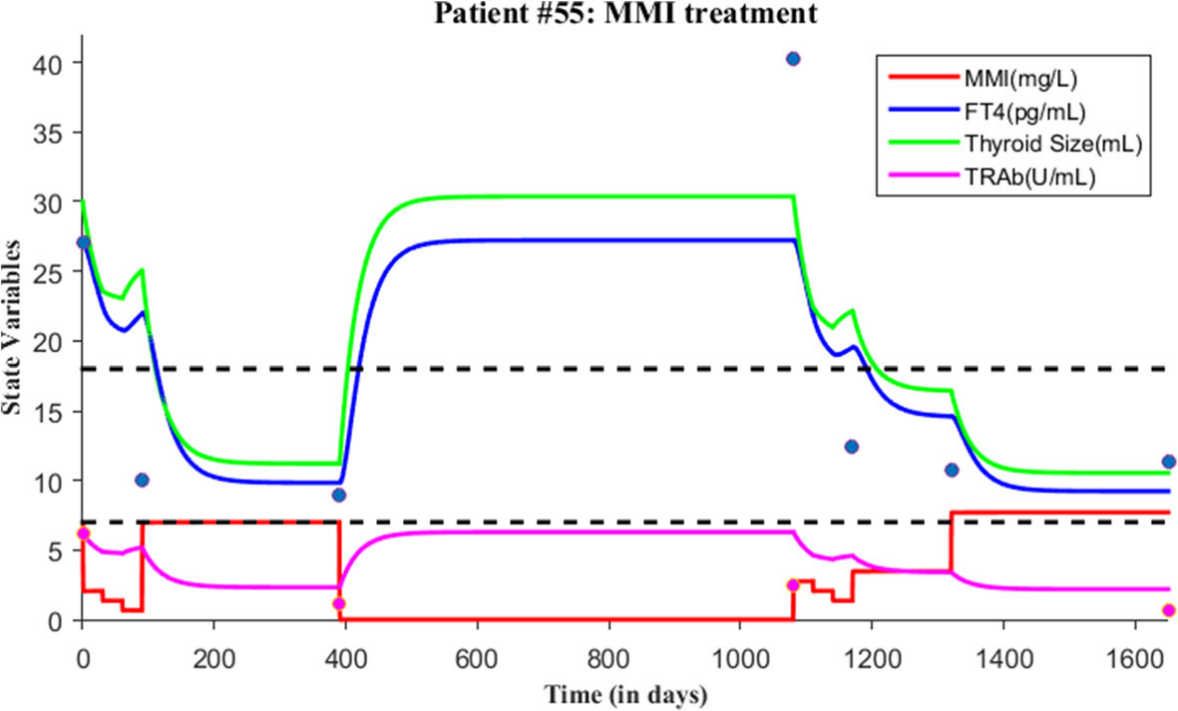
Two courses of MMI treatment is given for patient #55. Both FT4 and TRAb data is plotted with the simulated solutions. The root mean square error for the fit of FT4 and TRAb data is 7.3931 and 2.1257 respectively. Blue dots indicate FT4 levels while magenta dots indicate TRAb levels in the figure

Next, we simulate the model for patient #55 based on the above dosage information. For the 1st time period (i.e., 1 month), the value of *c*=7.01 and the initial value *x*_0_=20/3=6.7 mg/L, *y*_0_=27.01 pg/mL, *z*_0_=30 mL and *w*_0_=6.2 U/mL. For the 2nd time period (i.e., 1 month), the value of *c*=4.6726 and the initial value was the last point in the previous run or simulation. For the 3rd time period (i.e., 1 month), the value of *c*=2.3363 and the initial value was the last point in the previous run. For the 4th time period (i.e., 10 months), the value of *c*=23.3625 and the initial value was the last point in the previous simulation. For the 5th time period (i.e., no treatment for 23 months), the value of *c*=0.001 and the initial value was the last point in the previous simulation. Relapse of hyperthyroidism occurred so the second course of MMI treatment started. For the 6th time period (i.e., 1 month of loading dose), the value of *c*=9.3456 and the initial value was the last point in the previous run. For the 7th time period (i.e., 1 month), the value of *c*=7.01 and the initial value was the last point in the previous run. For the 8th time period (i.e., 1 month), the value of *c*=4.6726 and the initial value was the last point in the previous run. For the 9th time period (i.e., 5 months), the value of *c*=11.6817 and the initial value was the last point in the previous run. For the 10th time period (i.e., 11 months), the value of *c*=25.6996 and the initial value was the last point in the previous run. All parameter values are fixed in the simulation except *c* (see Table 3 and Fig. 16). To determine the accuracy of model fit to data, we calculated the root mean square error (RMSE) for the fit of FT4 and TRAb data, which is 7.3931 and 2.1257 respectively. The larger RMSE for FT4 and TRAb indicates a poor measure of accuracy so generating patient treatment schedule might not be reliable for patient #55. The larger RMSE can be due to a outlier present in patient data (see Fig. 16) and due to the lack of TRAb data.

## Results and discussions

When the treatment parameter *s*(*t*)=*c*=0 and the initial concentration of MMI *x*_0_=0, there is a unique steady state corresponding to hyperthyroidism, which is asymptotically stable. After the initiation of MMI treatment, that is *s*(*t*)=*c*>0 and *x*_0_>0, the hyperthyroidism steady state is moving on the monotonic decreasing function *w*=*Nz* towards subclinical hyperthyroidism and then euthyroidism. Once patients achieve euthyrodism, the maintenance dosage is prescribed to maintain FT4 values within the normal reference range. The value of treatment parameter *c* is determined in accordance with loading or maintenance dosages, treatment time period and body volume of patients. The initial concentration of MMI is estimated from the loading dosage of MMI.

One way to use the model is to run an experiment on clinical dosing amount and schedules. For example, by keeping the dosing MMI amount constant (say 5 mg) throughout the treatment period and varying the dosing schedule, say every 4 h for the 1st 130 days and/or every 8 h for the 2nd 130 days. We can actually check to see if that assumption ever results in hypothyroidism. Next, by keeping the dosing schedule constant say every day for 360 days, we can vary the dosing amount from high doses (say 30 mg) to low doses (say 5 mg) each day. After reaching the lowest dosage amount, restart the dosage amount from high to low doses again. In this way, we can actually check to see if that assumption results in subclinical hyperthyroidism, euthyroidism or hypothyroidism. Another way to use to model is to find the response rate of thyroid gland to different concentrations of MMI in the blood serum. Also, the model can be extended to account for cellular autoimmune responses, absorption rates from the gut to serum and drug toxicity.

## Conclusions

For Graves’ hyperthyroidism patients, we developed a patient-specific treatment model to describe the time course of FT4 levels after administration of Methimazole (MMI) and to control FT4 levels within the physiological range (7−18) pg/mL with an appropriate maintenance dosage schedule. Our primary interest is to explain the natural history of Graves’ hyperthyroidism MMI treatment, restore FT4 levels with an appropriate dosage amount and provide a better dosage schedule through the model. The model takes the form of a system of ordinary differential equations with four state variables, namely concentration of MMI (*x*) at time *t*, concentration of free thyroxine (*y*) at time *t*, concentration of TRAb (*w*) at time *t* and the functional size of thyroid gland (*z*) at time *t*. The model has total of thirteen positive parameters in which we fix twelve parameters for all runs except a treatment parameter *s*(*t*)=*c*. All fixed twelve parameters are determined from patients’ data and literature (see Table 3). The treatment parameter value *c* varies between patients, which in fact depends on amount of MMI orally taken, number of days of loading or maintenance dosages given, and body volume of the patient.

There is high variability among Graves’ hyperthyroid patients, making each patient unique so treatment with MMI is typically challenging. Right loading, maintenance dosage amount and schedule would help the patients control their free thyroxine levels within the normal reference range over time. Our model can be used for each patient to generate dosage amount and schedule which in turn may help prevent undershooting, overshooting and relapses. Basically, the model can predict when to discontinue the treatment which helps with decision-making on the amount of oral intake of MMI over time. With appropriate MMI dosage schedule, the clinical progression from hyperthyroidism → euthyroidism can be achieved effectively. We validated the model with data from four Graves’ hyperthyroidism patients. To measure the quality of prediction, we have calculated root mean square error for all of these four patients which can be cross-checked with the reference interval. The non-normalized root mean square error interval has been obtained for FT4 and TRAb using our patients’ data.

## Additional files

Additional file 1 Graves’ patients data. (XLSX 20 kb)

Additional file 2 RMSE interval estimation. (XLS 155 kb)
